# Supplementary material for: Epigenetic Alterations in DCIS Progression: What Can lncRNAs Teach Us?
Source: Int J Mol Sci. 2023 May 13;24(10):8733. doi: 10.3390/ijms24108733 (PMC10218364; doi:10.3390/ijms24108733)
Supplement: Supplementary file 1 [file ijms-24-08733-s001.zip › ijms-2355840-supplementary.pdf]

Supplementary files

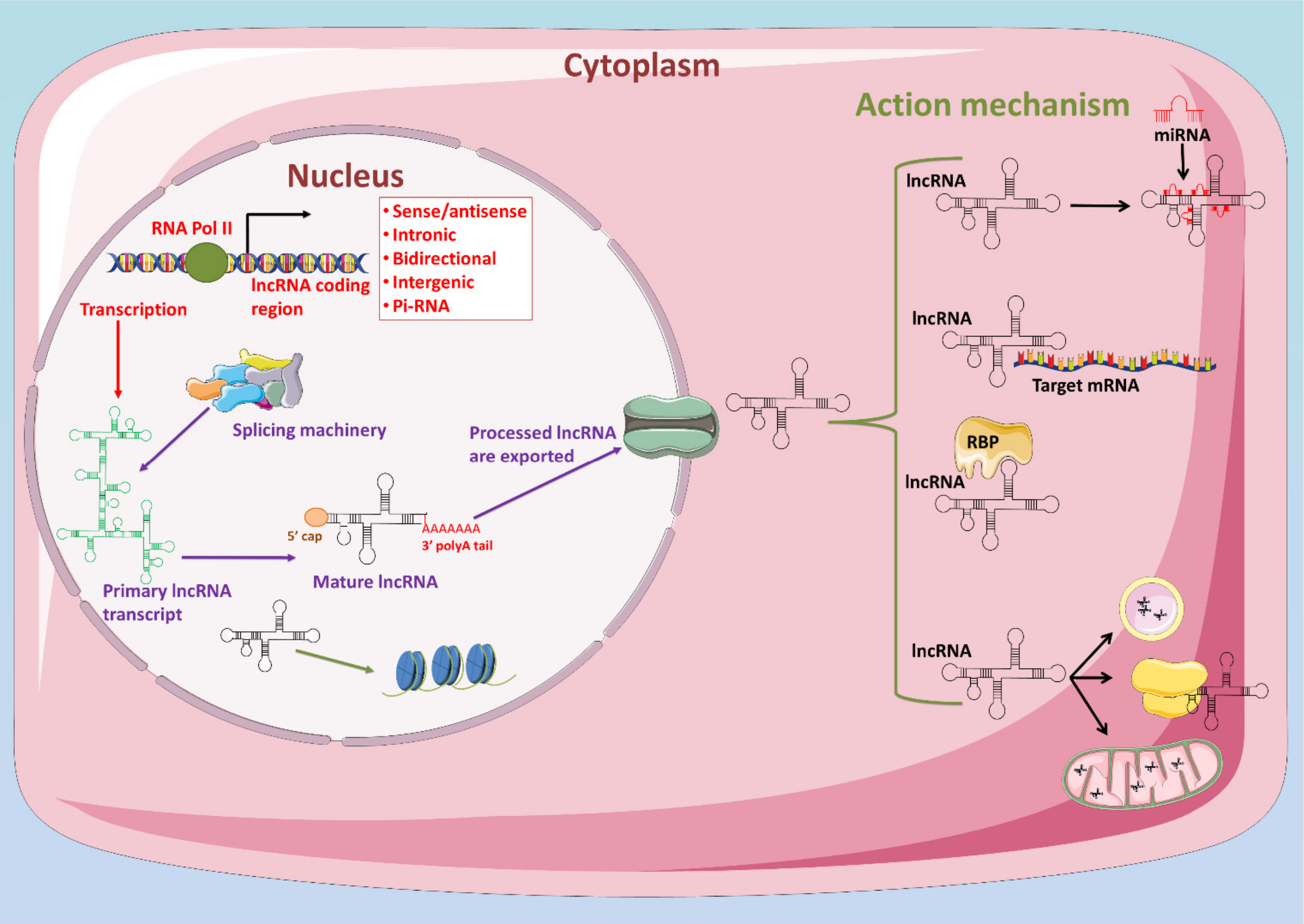

Figure S1. LncRNA biogenesis and function. LncRNAs are transcribed by RNA Polymerase II. Immature lncRNA molecules un-dergo splicing processing similar to messenger RNA (mRNA). Effective processing culminates in the formation of a mature lncRNA molecule containing a capped 5' end (5' UTR) and a polyadenylated 3' end (poly A tail). The processed lncRNA molecule is exported to the cytoplasm, where it can have different fates, many of which are relatively unknown. Mature lncRNAs can in-teract with and regulate other molecules, including RNA molecules (miRNAs and even mRNAs). Mature lncRNAs can also interact with diverse RNA-binding proteins (RBPs) and associate with ribosomes through 'pseudo' 5′ untranslated regions (UTRs). Several lncRNAs are sorted into mitochondria and can also be found in other organelles, such as exosomes; this recruitment of lncRNAs is accomplished by unknown mechanisms. LncRNAs that are inefficiently processed are retained in the nucleus. They can remain on chromatin andbind to chromatin-modifying complexes and direct them to a specific target, activating or repressing the target.

Table S1. Landscape regulation of lncRNA in DCIS

lncRNA	Modulation of molecular pathways and process	Premalignant phenotypic alteration	In vivo / in vitro alterations	Modulation of neighboring genes	Target gene	References
**HOTAIR**	Drug resistance; EMT, AP1, HIF1A; Autophagy	Growth; Invasion; Migration; Proliferation	Recurrence score; Increased in more aggressive DCIS lesions; Upregulated between normal breast and DCIS	N.D	N.D	[48, 52-55]
**LINC00885**	CDK6; MYC; ERBB; P53; EREG	Growth; Motility and migration; Cell proliferation	N.D	N.D	N.D	[59]
**BHLHE40-AS1**	IL-6 / STAT3	Increased invasive potential; Invasion; Migration	Immuno-permissive microenvironment; Disease progression; Upregulated between early-stage DCIS and IBC	N.D	N.D	[66]
**MALINC1**	Ap1; Cell adhesion; Tumor microenvironment remodeling; Extracellular matrix organization; Cell proliferation; Innate and adaptive immune response	N.D	Modulation of molecular pathways and process	N.D	N.D	[72, 73]
**RP11-379F4.4**	NF-kB	N.D	Potential indicator of DCIS-IBC progression	STAT	RARRES1	[88]
**RP11-465B22.8**	NF-kB	N.D	Potential indicator of DCIS-IBC progression	STAT	miR-200b; miR-200b	[88]

[*] N.D = Not Determined.
